# Relación entre la calidad del aire y el asma en habitantes de grandes altitudes, La Paz, Bolivia (3.600 m.s.n.m.)

**DOI:** 10.7705/biomedica.7155

**Published:** 2024-05-30

**Authors:** Lizeth Huanca-Laura, Marilyn Aparicio, Demetrio Jemio, Mariana Hurtado, Mayra Huanca, Alexis Chuquimia

**Affiliations:** 1 Unidad de Cambio Climático Ambiente y Salud, Instituto Boliviano de Biología de Altura, Facultad de Medicina, Universidad Mayor de San Andrés, La Paz, Bolivia Universidad Mayor de San Andrés Universidad Mayor de San Andrés La Paz Bolivia; 2 Unidad de Nefrología del Hospital de Clínicas, La Paz, Bolivia Hospital de Clínicas Hospital de Clínicas La Paz Bolivia; 3 Unidad de Flujo, Termometría y Electricidad, Instituto Boliviano de Metrología, La Paz, Bolivia Instituto Boliviano de Metrología Instituto Boliviano de Metrología La Paz Bolivia

**Keywords:** criterios de calidad del aire, material particulado, asma, espirometría, Air quality criteria, particulate matter, asthma, spirometry

## Abstract

**Introducción.:**

El asma es una enfermedad crónica que afecta a millones de personas en todo el mundo. La calidad del aire es uno de los factores clave que puede desencadenar los síntomas del asma.

**Objetivo.:**

Analizar la calidad del aire y su relación con el asma en habitantes de grandes altitudes en La Paz (Bolivia).

**Materiales y métodos.:**

Se desarrolló un estudio analítico, descriptivo y retrospectivo. Se recolectaron datos de pacientes con diagnóstico de asma en el Instituto Nacional del Tórax y en el Instituto Boliviano de Biología de Altura. Además, se monitoreó la calidad del aire y su material particulado en las estaciones de la “Red de monitoreo de la calidad del aire”.

**Resultados.:**

El 56,9 % de los casos fueron mujeres del Instituto Nacional del Tórax y el 45,7 % del Instituto Boliviano de Biología de Altura. En ambas instituciones, la media de edad fue de 47 años y los pacientes presentaban sobrepeso u obesidad. Se registraron incrementos de material particulado fino (PM_2,5_) en otoño, invierno y primavera, en 2014, 2016-2019 y en las cuatro estaciones del 2015. El material particulado inhalable grueso (PM_10_) se incrementó en otoño e invierno del 2014 al 2020, dentro de los límites establecidos. Se observó una asociación positiva y significativa entre la concentración de material particulado PM_2,5_ y los parámetros espirométricos de capacidad vital forzada, flujo espiratorio máximo y el porcentaje de reversión. La relación de partículas PM_10_ y los parámetros espirométricos de capacidad vital forzada, volumen espiratorio máximo en el primer segundo y flujo espiratorio máximo, también fue estadísticamente significativa.

**Conclusión.:**

Los casos de asma se presentaron en promedio a los 47 años y en personas con sobrepeso u obesidad. Se observó una asociación positiva entre el material particulado, PM_2,5_ y PM_10_, con los parámetros espirométricos, la cual fue más marcada con las partículas PM_2,5_.

## Abstract (en)

**Introduction.:**

Asthma is a chronic disease affecting millions of people around the world. Air quality is a major factor in triggering asthma symptoms.

**Objective.:**

To analyze air quality and asthma in high-altitude residents of La Paz, Bolivia.

**Materials and methods.:**

In this analytical, descriptive, and retrospective study, we collected data from patients diagnosed with asthma at the *Instituto Nacional del Tórax* and the *Instituto Boliviano de Biología de Altura*. In addition, air quality monitoring of particulate matter was carried out at the stations of the *Red de Monitoreo de la Calidad del Aire*.

**Results.:**

Women represented 56.9% of cases at the *Instituto Nacional del Tórax* and the *Instituto Boliviano de Biología de Altura*. In both institutions, the average age was 47 years and patients were overweight or obese. Increases in PM_2.5_ were recorded in autumn, winter and spring from 2014, 2016 to 2019 and 2015 in all four seasons. PM_10_ showed increases in autumn and winter from 2014 to 2020 within the established limits. We observed a positive and significant association between PM_2,5_ concentration and the spirometry parameters of forced vital capacity, peak expiratory flow^,^, and “reversibility percentage” or “bronchodilator response percentage”. The association of PM_10_ and forced vital capacity, forced expiratory volume in the first second, and peak expiratory flow, was also statistically significant.

**Conclusion.:**

Asthma cases occur on average at 47 years of age in overweight or obese people. We observed a positive association between particles PM_2,5_ and PM_10_ with spirometric parameters, stronger with particulate matter PM_2,5_.

El asma es una enfermedad crónica del sistema respiratorio que se caracteriza por la inflamación y el estrechamiento de las vías respiratorias, lo que dificulta la respiración y puede provocar episodios de sibilancias, tos y dificultad para respirar ^1^^-^^3^. La calidad del aire es un factor clave que puede influir en la exacerbación o aparición de los síntomas del asma ^4^^,^^5^.

La exposición a contaminantes atmosféricos como el dióxido de nitrógeno (NO_2_), el ozono (O_3_), el material particulado (PM_10_ y PM_2,5_) y otros compuestos químicos, puede desencadenar o empeorar los síntomas del asma en personas sensibles. Se han demostrado previamente asociaciones positivas entre contaminantes ambientales (NO_2_, O_3_, SO_2_ y PM_2,5_) y el uso de medicación de rescate, ya sea en entornos de contaminantes^,^ múltiples o exposición prolongada hasta de tres días ^6^. La exposición a alérgenos como el polen, los ácaros del polvo y los pelos de animales, también puede inducir síntomas de asma ^7^^,^^8^.

Las partículas en suspensión no están conformadas por un solo contaminante, sino que son una mezcla de muchas especies químicas. Es una combinación compleja de sólidos y aerosoles compuesta de pequeñas gotas de líquido, fragmentos sólidos secos y núcleos sólidos con recubrimientos líquidos ^6^. El material particulado PM_2,5_ y PM_10_ es transportado por el aire y procede de emisiones primarias (combustión de fósiles, desgaste de neumáticos) y partículas secundarias (nitratos y sulfatos), y se forman cuando los contaminantes reaccionan en la atmósfera. Las partículas PM_2,5_ son llamadas también “finas” mientras que las PM_10_ se definen como “gruesas^,^” ^9^. Las partículas finas pueden penetrar profundamente en los pulmones y son de mayor riesgo para la salud, ya que su inhalación puede afectar las zonas periféricas de los bronquiolos. El PM_10_ también puede inhalarse e ingresar hasta las vías respiratorias superiores ^4^.

Diferentes estudios demostraron el efecto negativo de la contaminación atmosférica sobre la salud humana ^10^^,^^11^. Entre ellos, el efecto de la contaminación del aire con alteraciones de la función pulmonar. Los valores altos de contaminación atmosférica según los estándares del calidad del aire establecidos por la *United States Enviromental Protection Agency* (EPA) alteran directamente a personas que padecen asma y otros tipos de enfermedades pulmonares o cardíacas ^12^.

La contaminación del aire por material particulado se asocia con problemas de salud, como incremento de la tos, sibilancias, afectación de la función pulmonar, ataques de asma, cardiopatías y muertes prematuras ^13^^,^^14^. Sin embargo, el material particulado no es un factor causal directo de enfermedad o mortalidad respiratoria aguda, sino un factor asociado que, en combinación con otros, produce un aumento de las enfermedades respiratorias ^11^^,^^15^.

Con la espirometría se evalúan las propiedades mecánicas del sistema respiratorio y es método de referencia para identificar obstrucciones del flujo aéreo que pueden resultar en asma, enfermedad pulmonar obstructiva crónica (EPOC), etc. ^16^. La espirometría permite medir la cantidad de aire que ingresa y egresa de los pulmones en función del tiempo, según el calibre de los bronquios, las propiedades elásticas de los pulmones y los músculos de la cavidad torácica ^16^.

En Bolivia, Cochabamba es la ciudad con mayor contaminación atmosférica causada por la actividad humana y la que muestra el mayor índice de contaminación de acuerdo con la Organización Mundial de la Salud (OMS). La “Red de monitoreo de la calidad de aire” de Cochabamba reportó que el 90 % de la contaminación aérea es se debe al parque automotor, ya sea de servicio particular o público ^17^.

Según la clasificación de altitudes, La Paz se encuentra en la categoría de ciudades con gran altitud (alto, igual a 1.500 a 3.500 m.s.n.m.; gran altitud o muy alto, de 3.500 a 5.500 m.s.n.m.; y extrema altitud, mayor de 5.500 m.s.n.m.) ^18^. Debido a sus características geográficas y meteorológicas, los niveles de contaminación en La Paz no revisten una problemática. Sin embargo, en la actualidad se observa contaminación moderadamente elevada o baja que igual puede producir efectos peligrosos sobre la salud de la población ^19^^-^^21^. La población de La Paz se encuentra sometida a una hipoxia hipobárica ambiental que puede producir efectos fisiológicos en el cuerpo humano, desde que nace hasta que se adapta a la altitud, como menor ventilación pulmonar, disminución del gradiente alvéolo-arterial de la concentración de oxígeno y aumento de la difusión alvéolo-capilar (Murillo-Jáuregui C, Romero C, Gonzales C, Alarcón AM, Aguilar M, Villena M. Hallazgos de función pulmonar en pacientes con EPOC a 3.600 m.s.n.m. en el Instituto Boliviano de Biología de Altitud. II Congreso Internacional de Medicina de la Altitud “Dr. Eduardo Aranda Torrelio”, 24 al 26 de febrero de 2016. La Paz, Bolivia. Disponible en: https://repositorio.umsa.bo/handle/123456789/10221).

Las condiciones urbanas y la calidad del aire son elementos importantes para las autoridades nacionales y locales, debido a los riesgos ambientales y sanitarios que deben tenerse en cuenta para mejorar la calidad de las ciudades, promover la protección de los ciudadanos frente a riesgos ambientales y generar oportunidades para mitigar la desigualdad en zonas menos favorecidas donde la carga ambiental tiende a ser mayor ^22^.

## Materiales y métodos

Se desarrolló un estudio analítico, descriptivo y retrospectivo, mediante la recolección de datos de pacientes con diagnóstico de asma atendidos en el Instituto Nacional del Tórax y el Instituto Boliviano de Biología de Altura, en el periodo 2014 a 2020.

Se revisaron las historias clínicas de los pacientes con diagnóstico de asma que contaban con espirometría, una prueba diagnóstica que se utiliza para evaluar la función pulmonar. En el caso del asma bronquial, la espirometría es un examen importante para determinar la gravedad de los síntomas y hacer su seguimiento, así como para evaluar el efecto del tratamiento. Esta prueba mide la cantidad y el flujo de aire que una persona puede exhalar, lo que ayuda a diagnosticar alguna obstrucción en las vías respiratorias. La ecuación de William Knudsen se utiliza para interpretar la espirometría y evaluar así la función pulmonar-^16^.

Con la espirometría se miden los siguientes parámetros.

*Capacidad vital forzada* (CVF). Es la cantidad de aire que se moviliza en una inspiración o espiración máxima forzada. Se expresa en mililitros y su valor normal es de 3.000 a 5.000 ml (según edad, altitud, sexo y raza); el resultado debe ser mayor de 80 % del valor teórico. La capacidad vital forzada puede disminuir en caso de enfermedades que afecten la capacidad pulmonar para expandirse o contraerse, como el asma, la enfermedad pulmonar obstructiva crónica y la fibrosis pulmonar.

*Volumen espiratorio máximo en el primer segundo* (FEV_1_). Es la cantidad de aire que se moviliza en el primer segundo de una espiración forzada. Es un flujo y no un volumen, de modo que puede expresarse en ml/s o como un porcentaje respecto a cifras teóricas. Su valor normal es mayor del 80 % del predicho.

*Índice de permeabilidad - cociente* FEV_1_ / FVC. Este aporta información sobre la cantidad del aire total espirado en el primer segundo. Es una tasa, por lo que suele expresarse como un porcentaje. El valor predicho normal es mayor del 75 al 80 %, sin embargo, en casos de obstrucción es del 70 %.

*Flujo espiratorio máximo* (FEM). Es la cantidad máxima de aire que se puede exhalar por segundo en una espiración forzada. Es un marcador de diagnóstico útil en las crisis asmáticas, en las cuales se emplea como valor predictivo de la gravedad del asma.

*Flujo espiratorio máximo en el 50 %* (FEF_50%_). Es la medición del flujo forzado en el 50 %. Evalúa la obstrucción de las vías aéreas superiores.

*Flujo espiratorio máximo en el 75 %* (FEF_75%_). En la altitud de La Paz, el FEF_75%_ tiene un valor menor a nivel del mar por la densidad del aire que es menor a mayor altura. El valor de referencia es del 70 %.

*Cambio significativo con broncodilatador:* Se considera como una mejoría mayor de 200 ml de la capacidad vital forzada o del 12 % respecto al valor basal del volumen espiratorio máximo en el primer segundo.

Al revisar la prueba de la espirometría de los pacientes con diagnóstico de asma, se valoró el índice de permeabilidad (FVC_1_/FVC) para determinar si había obstrucción. En caso afirmativo, se determinó si la obstrucción era central (FEF_50%_) o periférica (FEF_75%_). El valor normal en la altitud para FEF_50%_ es del 80 % y para FEF_75%_ es del 70 %. Posteriormente, se valoró el volumen espiratorio máximo en el primer segundo para determinar el grado de obstrucción (se tomaron en cuenta las recomendaciones de la *American Thoracic Society*). Se analizó la capacidad vital forzada para confirmar la ausencia de obstrucción y se utilizó la prueba de broncodilatación con 400 μg de salbutamol para evaluar si la obstrucción de los bronquios era reversible.

Se calculó el índice de masa corporal (IMC) de los pacientes, un indicador de la relación entre el peso y la altura utilizado para identificar sobrepeso u obesidad en los adultos. Se calcula dividiendo el peso de una persona en kilogramos entre el cuadrado de su talla en metros (kg/m^2^). Una persona con sobrepeso puede tener un IMC igual o superior a 25 kg/m^2^, mientras que una obesa puede tener un IMC igual o superior a 30 kg/m^2^.

**Figura 1:**
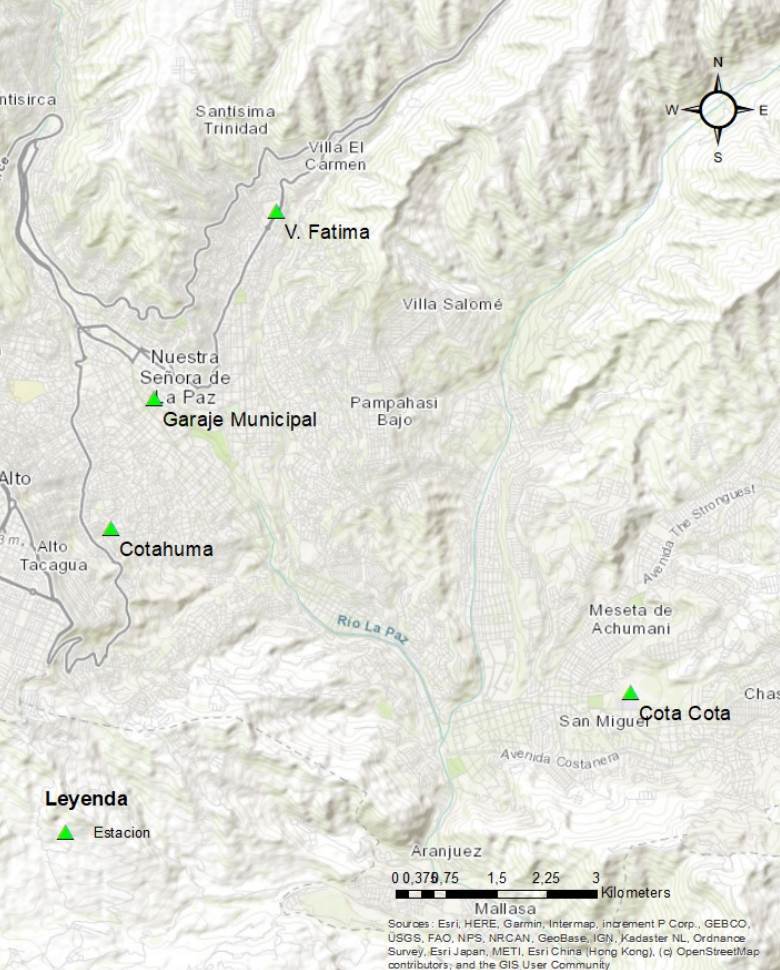
Estaciones de monitoreo en la ciudad de La Paz

Se monitoreó la calidad de aire mediante la medición del material particulado (PM_2,5_ y PM_10_) en estaciones de la “Red de monitoreo de la calidad del aire” ^,^de La Paz (Red MoniCA) (figura 1).

Fuentes: Gobierno Autónomo Municipal de La Paz, Secretaría Municipal de Gestión Ambiental, Unidad de Servicios Ambientales, Red de Monitoreo de Calidad del Aire (MoniCA)

El material particulado PM_2,5_ fue recolectado por un método activo durante 24 horas en una sola estación:^,^ “Garaje Municipal” en La Paz. El material particulado PM_10_ fue recolectado por dos métodos: el automático continuo y el activo, en las estaciones Cota Cota, Villa Fátima, Cotahuma, Garaje Municipal y Tránsito, en La Paz.

Para la correlación de los datos de la calidad de aire, se revisaron las normas bolivianas y las de la OMS sobre los límites permitidos del material particulado (PM_2,5_ y PM_10_). Se elaboró la base de datos con la información recolectada y se ^,^procesó la información. Posteriormente, se analizaron los datos con el paquete estadístico Stata 14.

## Resultados

### Característica de la población atendida en el Instituto Nacional del Tórax

En el Instituto Nacional del Tórax, del 2014 a 2020, se atendieron 3.530 pacientes: 942 con diagnóstico de asma bronquial y 2.588 con enfermedad pulmonar obstructiva crónica. El 56,9 % fueron mujeres y el 43,1 % hombres, con un promedio de edad de 47 años. El 44,5 % de la población tenía sobrepeso y, el 29,8 %, obesidad. La mayoría de los pacientes tenían ocupaciones varias, seguidos por aquellos dedicados a labores de casa, comerciantes u oficinistas (cuadro 1).

**Cuadro 1:** Características de la población asmática del Instituto Nacional del Tórax (N = 942)

Característica		n (%)	
Edad (media ± desviación estándar)		47	(16,73)
Sexo			
	Femenino	536	(56,9)
	Masculino	406	(43,1)
Índice de masa corporal			
	Normal	242	(25,7)
	Sobrepeso	419	(44,5)
	Obesidad	281	(29,8)
Estado civil			
	Soltero	513	(54,5)
	Casado	3,9	(41,5)
	Viudo	26	(2,8)
	Divorciado	12	(1,3)
Ocupación			
	Labores de casa	280	(29,7)
	Minero	15	(1,6)
	Agricultor	44	(4,7)
	Carpintero	8	(0,9)
	Comerciante	124	(13,2)
	Oficinista	65	(6,9)
	Jubilado	14	(1,5)
	Otros	392	(41,6)

### *Monitoreo de material particulado PM*_*2,5*_ *del 2014 al 2020 (método activo)*

**Figura 2:**
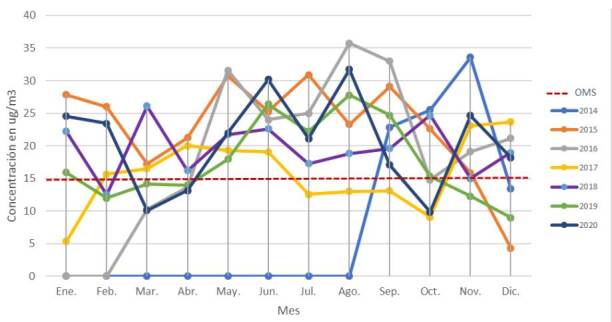
Promedio mensual de PM_2,5_ del 2014 al 2020

Se identificó material particulado PM_2,5_ en concentraciones superiores a las establecidas por la OMS en el nuevo informe del 2021 (15 μg/m^3^), en los siguientes periodos: primavera (septiembre a noviembre) del 2014, en las cuatro estaciones (enero a noviembre) del 2015, en otoño, invierno y primavera (mayo a octubre) del 2016, en otoño y primavera (febrero a junio y noviembre y diciembre) del 2017, en otoño, invierno y primavera (marzo a diciembre) del 2018, en otoño, invierno y primavera (mayo a octubre) del 2019 y en las cuatro estaciones (enero y febrero; mayo a septiembre; noviembre y diciembre) del 2020 (figura 2 y 3).

Fuentes: Gobierno Autónomo Municipal de La Paz, Secretaría Municipal de Gestión Ambiental, Unidad de Servicios Ambientales, Red de Monitoreo de Calidad del Aire (MoniCA)

* Línea discontinua en rojo: límites establecidos por la Organización Mundial de la Salud (OMS) según el nuevo informe del 2021 (PM_2,5_ = 15 μg/m^3^ en 24 horas)

* Los valores nulos (cero) de concentración no se midieron.

**Figura 3:**
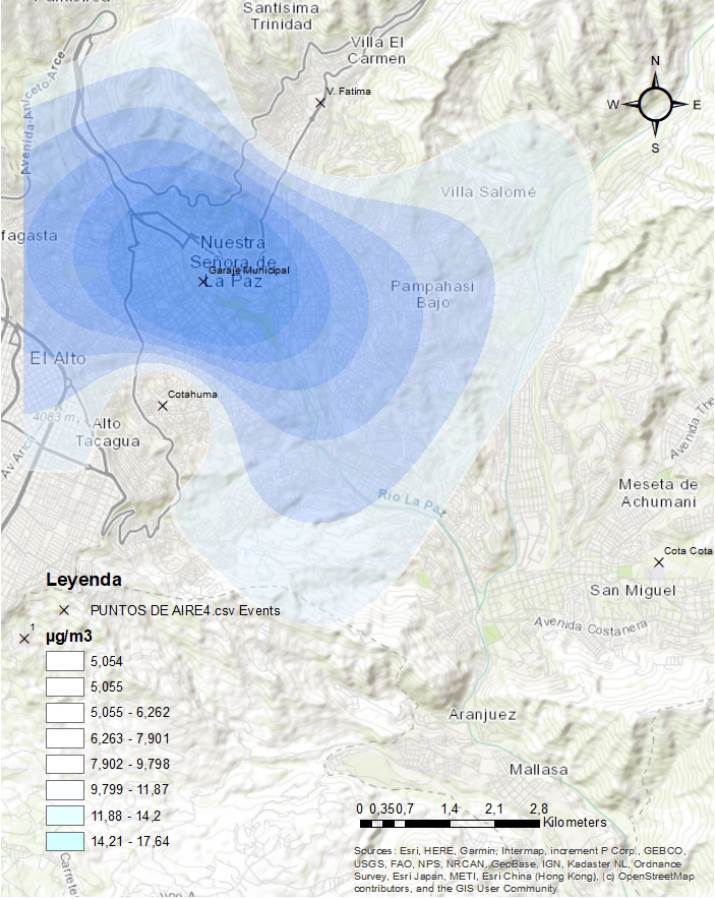
Calidad de aire con material particulado fino (≤ 2,5 μm) del 2019

Fuentes: Gobierno Autónomo Municipal de La Paz, Secretaría Municipal de Gestión, Ambiental, Unidad de Servicios Ambientales, Red de Monitoreo de Calidad del Aire (MoniCA)

Límites establecidos por la Organización Mundial de la Salud según el nuevo informe del 2021 (PM_2,5_ = 15 μg/m^3^ en 24 horas).

* Los valores nulos (cero) de concentración no se midieron.

### *Monitoreo de material particulado PM*_*10*_ *del 2014 al 2020 (método automático continuo)*

**Figura 4:**
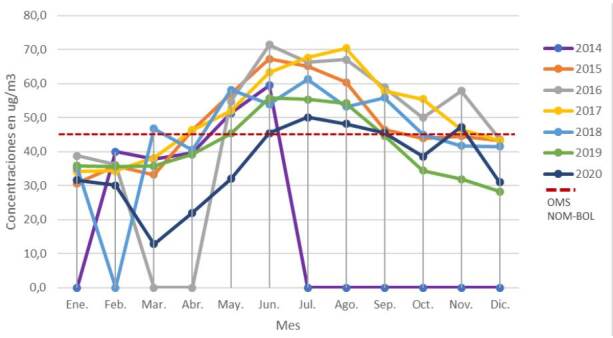
Promedio mensual de material particulado grueso (> 2,5 y ≤ 10) del 2014 al 2020

Se midieron concentraciones de material particulado PM_10_ superiores a los límites establecidos por la OMS en el nuevo informe del 2021 (45 μg/m^3^), en los siguientes periodos: en otoño e invierno (mayo y junio) del 2014, en otoño e invierno (de abril a septiembre) del 2015, en invierno y primavera (mayo a noviembre) del 2016, en otoño, invierno y primavera (abril a noviembre) del 2017, en invierno y primavera (mayo a septiembre) del 2018, en invierno (junio hasta agosto) del 2019 y finalmente, en invierno y primavera (junio a septiembre) del 2020 (figuras 4 y 5).

Fuentes: Gobierno Autónomo Municipal de La Paz, Secretaría Municipal de Gestión Ambiental, Unidad de Servicios Ambientales, Red de Monitoreo de Calidad del Aire (Red MoniCA)

* Línea discontinua en rojo: límites establecidos por la Organización Mundial de la Salud según el nuevo informe del 2021 (PM_10_ = 45 μg/m^3^ en 24 horas)

* Los valores nulos (cero) de concentración no se midieron.

**Figura 5:**
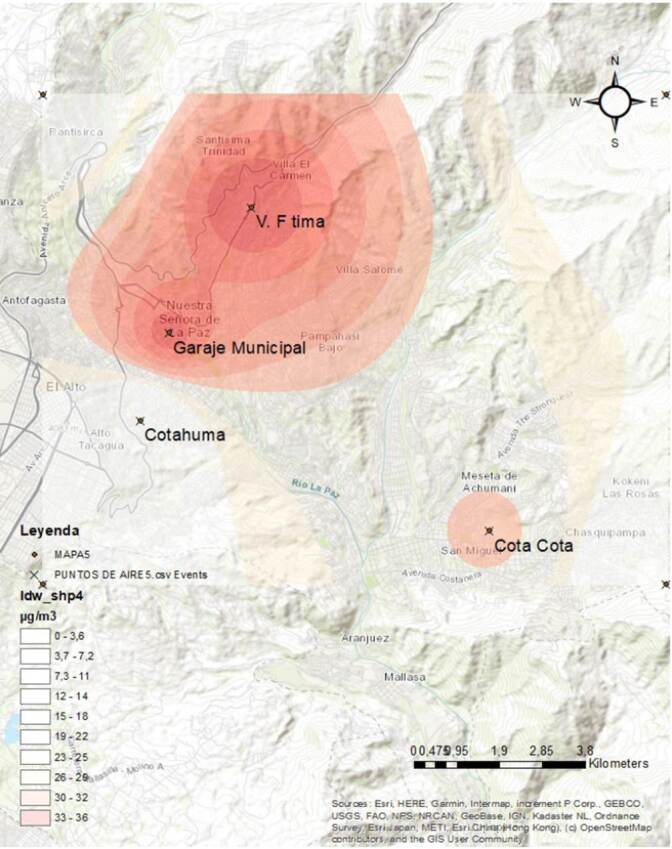
Calidad de aire con material particulado grueso (PM_10_) en el 2015

Fuentes: Gobierno Autónomo Municipal de La Paz, Secretaría Municipal de Gestión Ambiental, Unidad de Servicios Ambientales, Red de Monitoreo de Calidad del Aire (Red MoniCA).

Límites establecidos por la Organización Mundial de la Salud según el nuevo informe del 2021 (PM_10_ = 45 ug/m^3^ en 24 horas)

### Características de la población atendida en la Unidad de Fisiología y Fisiopatología Respiratoria del Instituto Boliviano de Biología de Altura

En la Unidad de Fisiología y Fisiopatología Respiratoria del Instituto Boliviano de Biología de Altura, se atendieron 8.401 pacientes del 2014 al 2019, de los cuales 709 tenían diagnóstico de asma. Se observó que el 45,7 % fueron mujeres y el 54,3 % hombres, con una media de edad de 54 años. La mayoría de la población tenía sobrepeso u obesidad, y se dedicaba a ocupaciones varias, seguida por aquellos con labores de casa, oficinistas y jubilados (cuadro 2).

**Cuadro 2:** Características de la población asmática de la Unidad de Fisiología y Fisiopatología Respiratoria del Instituto Boliviano de Biología de Altura (N = 709)

Característica		n (%)
Edad (media ± desviación estándar)		54 (17,9)
Sexo		
	Femenino	324 (45,7)
	Masculino	385 (54,3)
Índice de masa corporal		
	Normal	193 (27,2)
	Sobrepeso	282 (39,8)
	Obesidad	234 (33)
Ocupación		
	Labores de casa	159 (22,4)
	Minero	76 (10,7)
	Agricultor	23 (3,2)
	Carpintero	16 (2,3)
	Comerciante	75 (10,6)
	Oficinista	120 (16,9)
	Jubilado	113 (15,9)
	Otros	296 (53,7)

### Características de las pruebas funcionales respiratorias en la población asmática del Instituto Boliviano de Biología de Altura

Se revisaron las pruebas funcionales espirométricas y se observó que, en promedio, el índice de permeabilidad antes del broncodilatador fue del 67 % y, después del broncodilatador, fue del 74 %. La capacidad vital forzada fue del 108 % en promedio antes del broncodilatador y del 118 % después del broncodilatador. El flujo espiratorio forzado máximo 50 % fue de 46 % antes del broncodilatador y del 68 % después del broncodilatador; en el flujo espiratorio forzado máximo 75 %, se reportó un promedio de 50 % posterior al broncodilatador. El promedio del flujo espiratorio máximo general antes del broncodilatador fue del 86 % y después del broncodilatador fue del 100 %. El promedio de reversión fue mayor del 12 % después del broncodilatador.

### Correlación entre la calidad de aire de La Paz y las espirometrías de los asmáticos atendidos en la Unidad de Fisiología y Fisiopatología Respiratoria del Instituto Boliviano de Biología de Altura

La correlación entre el material particulado PM_2,5_ y la capacidad vital forzada, el flujo espiratorio máximo y el porcentaje de reversión posterior al broncodilatador, fue positiva y estadísticamente significativa (*p* < 0,05), esto indica que, cuanto mayor sea la concentración de partículas PM_2,5_, más se altera la capacidad vital forzada y, también, el flujo espiratorio máximo. El FEF_50%_ tiene una correlación positiva pero sin significancia estadística, mientras que el FEF_75%_ presentó una correlación negativa, también sin significancia estadística.

La correlación del material particulado PM_10_ con las pruebas espirométricas fue positiva y estadísticamente significativa con la capacidad vital forzada, el volumen espiratorio máximo en el primer segundo y el flujo espiratorio máximo. Los parámetros FEF_50%_ y FEF_75%_tuvieron una correlación positiva con el porcentaje de reversión, posterior al broncodilatador, pero sin significancia estadística. Finalmente, la única prueba que tuvo una correlación negativa con el material particulado PM_2,5_ y PM_10_ fue el índice de permeabilidad (cuadro 3).

**Cuadro 3:** Asociación de las concentraciones del material particulado PM_2,5_ y PM_10_ con los resultados de las pruebas es^,^ pirométricas de los pacientes asmáticos, aplicadas según las normas del Instituto Boliviano de Biología de Altura

Característica		Rho	p
PM_2,5_			
	FEV-1/FVC	-0,39	0,001*
	FVC	0,26	0,02*
	FEF 50%	0,02	0,9
	FEF 75%	-0,11	0,37
	FEV-1	0,16	0,17
	FEM	0,33	0,001*
	Reversión	0,35	0,001*
**PM**_10_			
	FEV-1/FVC	-0,07	0,56
	FVC	0,22	0,05*
	FEF 50%	0,17	0,14
	FEF 75%	0,09	0,46
	FEV-1	0,27	0,01*
	FEM	0,24	0,04*
	Reversión	0,06	0,56

FEV-1: volumen espiratorio máximo en el primer segundo; FVC: capacidad vital forzada; FEF: flujo espiratorio forzado máximo; FEM: flujo espiratorio máximo

* Valor p<0,05

## Discusión

En este estudio se analizó la relación entre la calidad del aire y el asma en habitantes de gran altitud de la ciudad de La Paz (Bolivia). Los resultados reportaron la correlación positiva, estadísticamente significativa, del material particulado PM_2,5_ con la capacidad vital forzada, el flujo espiratorio máximo y el porcentaje de^,^ reversión después del broncodilatador. La correlación de las partículas PM_10_ y las pruebas espirométricas fue positiva y estadísticamente significativa con los parámetros de capacidad vital forzada, volumen espiratorio máximo en el primer segundo y flujo espiratorio máximo.

El índice de permeabilidad o cociente FEV-1/FVC se encuentra relacionado con la cantidad del aire total espirado en el primer segundo: el valor normal es mayor de 70 % ^16^. Sin embargo, en el presente trabajo se reportó un promedio disminuido del 67 % antes del broncodilatador y del 74 % después. Esta correlación negativa puede deberse a diferentes factores del paciente (como peso, talla, etc.) y contrasta con el estudio del 2018 que mostró una asociación positiva entre el cociente FEV1/FVC en pacientes asmáticos y las partículas PM_10_ tras una exposición aguda en primavera ^23^.

El FEV-1 es la cantidad de aire que se moviliza en el primer segundo de una espiración forzada; el valor normal es mayor del 80 % ^16^. En este trabajo se reportó un promedio del 118 % posterior al broncodilatador, considerado dentro de lo normal, y una correlación positiva con el material particulado PM_2,5_ y PM_10_.

El flujo espiratorio máximo es uno de los mejores marcadores para el diagnóstico de asma y crisis asmáticas. Este reporte mostró una disminución del promedio posterior al broncodilatador y evidenció una correlación positiva de esta prueba con las partículas PM_2,5_ y PM_10_, lo que indica que, a mayor concentración de material particulado, mayor será la exacerbación de las pruebas espirométricas de los pacientes con asma.

Los valores de FEF_50%_ y FEF_75%_ en gran altitud son más bajos que a nivel del mar. El FEF_50%_ es indicador de obstrucción central (80 % valor normal en la altitud) mientras que el FEF_75%_señala obstrucción periférica (70 % valor normal en la altitud) ^16^ (Murillo Jáuregui C, Romero C, Gonzales C, Alarcón AM, Aguilar M, Villena M. Hallazgos de función pulmonar en pacientes con EPOC a 3.600 m.s.n.m. en el instituto Boliviano de Biología de Altura. II Congreso Internacional de Medicina de la Altura “Dr. Eduardo Aranda Torrelio” 24 al 26 de febrero de 2016. La Paz, Bolivia. Disponible en: https://repositorio.umsa.bo/handle/123456789/10221).

En este trabajo se observó una disminución de los valores FEF_50%_ y FEF_75%_ respecto a los normales. Sin embargo, el porcentaje de reversión después del salbutamol fue mayor del 12 % en los pacientes asmáticos. Estos datos apoyan la asociación positiva, estadísticamente significativa, del porcentaje de reversión con el material particulado PM_2,5_.

En este sentido, los contaminantes ambientales pueden agravar diferentes enfermedades respiratorias como el asma ^24^^-^^26^. Un estudio de series de tiempo reportó una correlación positiva entre la contaminación por material particulado y el incremento de pacientes internados por asma en los servicios hospitalarios ^24^.

El material particulado tiene un impacto negativo porque se deposita sobre las vías respiratorias de las personas asmáticas y provoca inflamación directa, edema de las mucosas y citotoxicidad ^27^. Los resultados de este trabajo sustentan esta información dada la correlación del flujo espiratorio máximo y los materiales particulados PM_2,5_ y PM_10_.

Otros reportes informaron la asociación aguda entre partículas PM_2,5_ y la exacerbación del asma en pacientes con diagnóstico previo de obesidad ^28^. Estos resultados son congruentes con los del presente estudio, donde se observó que la mayoría de la población tenía sobrepeso u obesidad, y cuya ocupación más frecuente fue la de comerciante. Investigaciones previas han reportado que los comerciantes que trabajan en la vía pública son un grupo vulnerable a la contaminación del aire y la elevación de la temperatura ambiental asociada con la disminución de la presión barométrica ^29^.

Respecto al uso de los servicios de salud en los casos de exacerbación del asma y los niveles de material particulado PM_10_ en zonas rurales de Estados Unidos, James *et al*. estimaron que, por cada aumento de 15 μg/m^3^ de PM_10_, los ingresos hospitalarios de los pacientes con asma se incrementaban un 3 % ^30^. En contraste, en el presente estudio se reporta un mayor ingreso por consulta neumológica externa (82 %), seguido por emergencias (14 %).

La calidad del aire en la ciudad de La Paz mostró mayores concentraciones de partículas PM_2,5_ en el 2014, en los meses de octubre a noviembre (primavera) en los que se incrementan las temperaturas; y en el 2016, en los meses de mayo, agosto y septiembre (fin del invierno e inicio de la primavera). Las menores concentraciones de contaminantes se obtuvieron en épocas de lluvia.

En el 2013, la evaluación de la calidad del aire en la ciudad de La Paz mostró concentraciones altas de PM_10_ en las épocas secas y no en las de lluvias ^31^. Sin embargo, en el presente trabajo se observaron concentraciones altas de PM_10_ en 2016 y 2017, en los meses de invierno, que son considerados como época fría y seca.

En los meses de enero y febrero; mayo a septiembre; noviembre y diciembre del 2020, PM_2,5_ tuvo concentraciones altas, mientras que el resto de los meses estuvo dentro de los parámetros establecidos por la OMS. El pico observado coincide con el inicio de la cuarentena en Bolivia. Por el contrario, a lo largo del 2020, las concentraciones del material particulado PM_10_ estuvieron dentro de los límites establecidos. Se considera que la cuarentena provocó una reducción drástica de las emisiones de contaminantes en las ciudades más pobladas ^32^.
